# Pirin is a prognostic marker of human melanoma that dampens the proliferation of malignant cells by downregulating *JARID1B/KDM5B* expression

**DOI:** 10.1038/s41598-023-36684-2

**Published:** 2023-06-12

**Authors:** Cristina Penas, Yoana Arroyo-Berdugo, Aintzane Apraiz, Javier Rasero, Iraia Muñoa-Hoyos, Noelia Andollo, Goikoane Cancho-Galán, Rosa Izu, Jesús Gardeazabal, Pilar A. Ezkurra, Nerea Subiran, Carmen Alvarez-Dominguez, Santos Alonso, Anja K. Bosserhoff, Aintzane Asumendi, María D. Boyano

**Affiliations:** 1 Department of Cell Biology and Histology, Faculty of Medicine and Nursing, UPV/EHU, 48940 Leioa, Spain; 2 Biocruces Bizkaia Health Research Institute, 48903 Barakaldo, Spain; 3 Department of Psychology, Carnegie Mellon University, Pittsburg, PA 15213 USA; 4 Department of Physiology, Faculty of Medicine and Nursing, UPV/EHU, 48940 Leioa, Spain; 5 Department of Pathology, Basurto University Hospital, 48013 Bilbo, Spain; 6 Department of Dermatology, Basurto University Hospital, 48013 Bilbo, Spain; 7 Department of Dermatology, Cruces University Hospital, 48903 Barakaldo, Spain; 8 MEDONLINE Multidisciplinary Research Group, Faculty of Health Sciences and Faculty of Education, International University of La Rioja, 26006 Logroño, Spain; 9 Department of Genetics, Physical Anthropology and Animal Physiology, Faculty of Science and Technology, UPV/EHU, 48940 Leioa, Spain; 10 Institute of Biochemistry, Friedrich-Alexander University of Erlangen-Nürnberg, 91054 Erlangen, Germany; 11 Comprehensive Cancer Center (CCC) Erlangen-EMN, 91054 Erlangen, Germany

**Keywords:** Cancer, Cell biology, Molecular biology, Biomarkers, Molecular medicine, Oncology, Risk factors

## Abstract

Originally considered to act as a transcriptional co-factor, Pirin has recently been reported to play a role in tumorigenesis and the malignant progression of many tumors. Here, we have analyzed the diagnostic and prognostic value of Pirin expression in the early stages of melanoma, and its role in the biology of melanocytic cells. Pirin expression was analyzed in a total of 314 melanoma biopsies, correlating this feature with the patient’s clinical course. Moreover, *PIR* downregulated primary melanocytes were analyzed by RNA sequencing, and the data obtained were validated in human melanoma cell lines overexpressing *PIR* by functional assays. The immunohistochemistry multivariate analysis revealed that early melanomas with stronger Pirin expression were more than twice as likely to develop metastases during the follow-up. Transcriptome analysis of *PIR* downregulated melanocytes showed a dampening of genes involved in the G1/S transition, cell proliferation, and cell migration. In addition, an in silico approach predicted that *JARID1B* as a potential transcriptional regulator that lies between *PIR* and its downstream modulated genes, which was corroborated by co-transfection experiments and functional analysis. Together, the data obtained indicated that Pirin could be a useful marker for the metastatic progression of melanoma and that it participates in the proliferation of melanoma cells by regulating the slow-cycling *JARID1B* gene.

## Introduction

Melanocytes are neural crest-derived cells principally located in the basal layer of the epidermis of the skin, where they remain under the strict control of cells like keratinocytes. Under normal homeostatic conditions, melanocyte proliferation occurs only after they are stimulated by paracrine factors secreted from keratinocytes^1^. By contrast, uncontrolled proliferation may be provoked by mutations, such as BRAFV600E, often detected in both benign (nevus) and malignant melanocytic lesions^2^. Nevertheless, mammalian cells possess mechanisms to limit excess proliferation and protect themselves from malignant transformation, such as oncogene-induced senescence (OIS). Therefore, melanocytes exhibiting oncogenic mutations must first bypass OIS for successful malignant progression and then, they must acquire the ability to spread. From this point, several cellular events are involved, such as a second boost of proliferation, a pseudo-epithelial-to-mesenchymal transition (EMT) and migration^3,4^. In this melanocytic context, a protein known as Pirin has been associated with the inhibition of senescence, although little is known about the mechanisms underlying this effect^5^.

The Pirin protein is a member of the cupin superfamily proposed to play a role in cancer. It was first described as a ubiquitously expressed nuclear protein with a putative transcriptional co-factor function^6^, which was soon reinforced when it was shown to interact with the B-cell lymphoma 3-encoded protein (BCL-3) in a yeast two-hybrid-system^7^. Several studies then revealed the implication of Pirin in a wide range of processes, including cell cycle control^8–10^, inflammatory responses^11,12^, migration and the EMT^13–17^. Moreover, Pirin inhibition has been related to weaker migratory capacity of melanoma cells^13^, and it has been proposed to inhibit cellular senescence^5^ and to serve as a malignant biomarker^18^.

Here we have analyzed the diagnostic and prognostic value of Pirin in early-stage melanoma biopsies (stages I and II according to the AJCC 8th edition^19^), as well as its role in the biology of primary melanocytes and malignant melanoma cells using RNA-seq and a functional analysis. Our results suggest that Pirin expression could represent a good prognostic marker and that it is involved in melanoma cell proliferation through the modulation of *JARID1B*.

## Results

### Pirin expression and the Breslow thickness in melanoma biopsies is correlated with the metastatic progression of melanoma

The expression of the Pirin protein in human melanocytic lesions was analyzed by immunohistochemistry on histological sections from 75 nevi and 239 melanomas, and correlated to the clinical data from the nevus and melanoma patients (Table 1). Regarding the tumor stage, 14% of melanomas were diagnosed in situ (n = 34), 71% as early I and II stages (n = 171), and 15% in advanced III and IV melanoma stages (n = 34). Moreover, 62% of the patients included in this study remained disease-free during the follow-up, while 38% developed metastasis. Indeed, of the early I and II stage melanoma patients, 33% developed metastasis during the follow-up (n = 57 of 171). Regarding the histopathological analysis of the nevi, most of them were intradermal nevi, although some compound and dysplastic nevi were also included. The survival of these patients was 100%.

**Table 1:** Clinical and pathological data from nevi and melanoma patients.

	N (%)
NEVUS	75
Age at diagnosis in years (range)	56 (24–78)
Sex	
Male	28 (37)
Female	47 (63)
MELANOMAS	239
Age at diagnosis in years (range)	57 (23–87)
Sex	
Male	131 (55)
Female	108 (45)
Localization	
Head and neck	43(18)
Trunk	74(31)
Upper limb	24 (01)
Lower limb	69 (29)
Acral	21 (9)
Others	5 (2)
ND	3 (1)
Histological subtype	
SSM	102 (43)
NM	53(22)
ALM	21(9)
LMM	9(4)
LM	3 (1)
Others	12 (5)
ND	39 (16)
AJCC stage at diagnosis	
In situ	34 (14)
IA	46 (19)
IB	54 (23)
IIA	32 (13)
IIB	15 (6)
IIC	24 (10)
IIIA	9 (4)
IIIB	11 (5)
IIIC	6 (3)
IV	8 (3)
Disease evolution	
Disease-free	147 (62)
Metastasis	92 (38)

*SSM* superficial spread melanoma, *NM* nodular melanoma, *ALM* acral lentigo melanoma, *LMM* lentigo malignant melanoma, *LM* lentigo melanoma.

**Figure 1:**
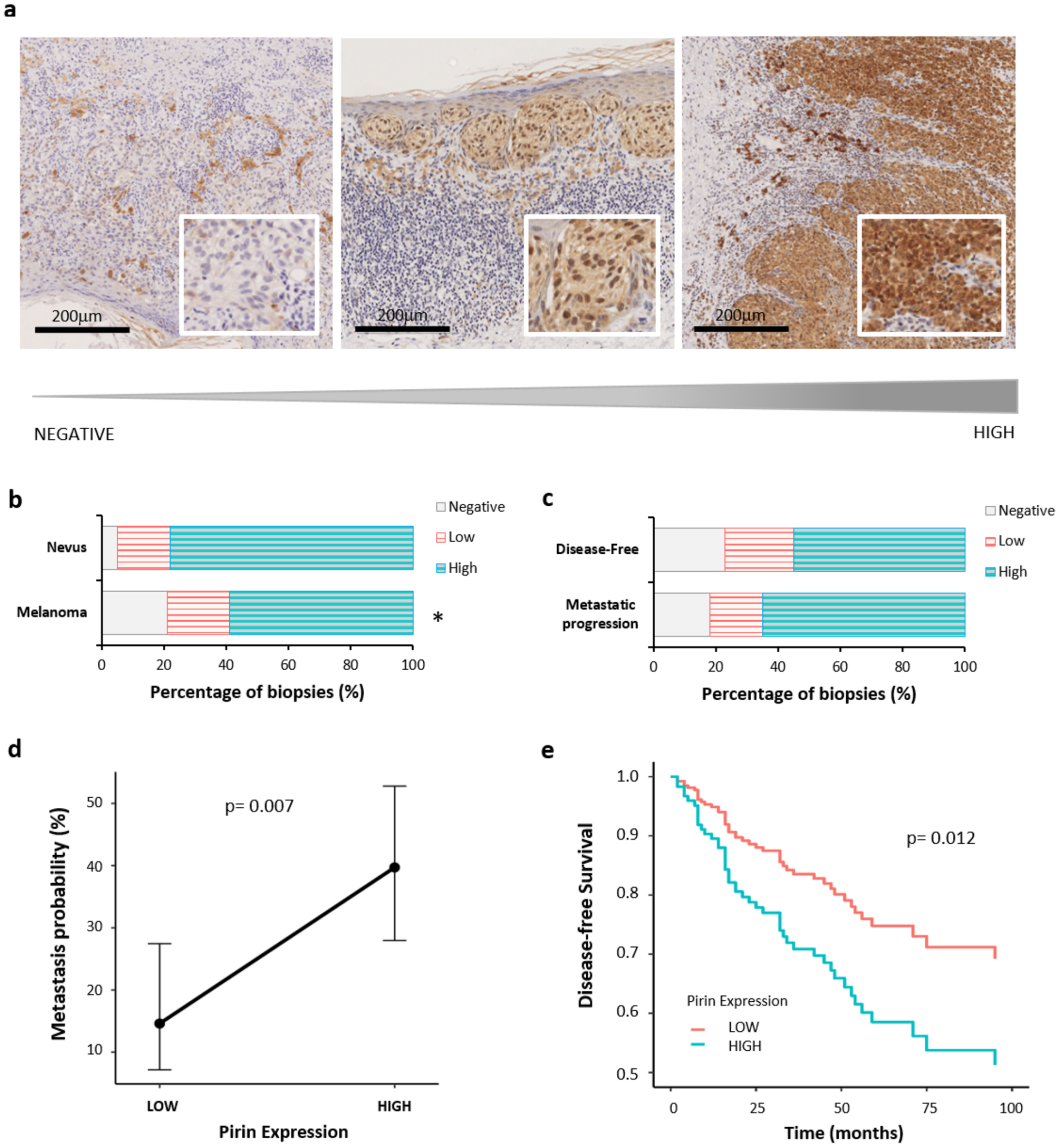
Pirin expression in FFPE biopsies from melanoma patients. (**a**) Immunostaining for Pirin in three representative melanoma biopsies (negative, low and high). (**b**) Comparative analysis of the proportion of cases with each level of Pirin expression between nevi and melanoma cases. (**c**) Comparative analysis of patients with early stage melanomas (I and II stages, AJCC 8th) who remained disease-free or developed metastasis during the follow-up and the proportion of cases with each level of Pirin expression. (**d**) The marginalized metastatic probability with respect to Pirin expression (Low and High) from a Logistic Regression model, which also included the Breslow Index, age and sex as co-variates. (**e**) Adjusted survival curves in terms of Pirin expression using a Cox Proportional-Hazard model: *p < 0.05.

Two independent pathologists analyzed these samples and the levels of Pirin expression was classified as negative, low or high (Fig. 1a). When comparing Pirin expression in nevi and melanomas, we first observed that 80% of the nevi biopsies were strongly stained for Pirin, which was significantly more than in the cohort of melanoma samples, in which strong Pirin expression was seen in 60% of the histological sections (*p* < 0.05: Fig. 1b).

The usefulness of Pirin expression as an early prognostic marker was evaluated, only using the biopsies from early stage melanoma patients (stages I and II, according to AJCC 8th) and classifying them according to their clinical progression (metastasis or not: Fig. 1c). We did not observe a direct association between Pirin expression and the patients who remained disease-free or those who developed metastases during the follow-up (Cochran-Armitage χ^2^ = 1.372, *p* = 0.271). However, since our data from early primary melanomas was heterogeneous, one might expect that other potential risk factors might be masking the association between Pirin expression and the probability of metastasis. For example, both age and Breslow depth appeared to be statistically higher in the metastatic group (Z = − 3.965, *P* < 0.001) than in the disease-free group (Z = − 8.0526, *P* < 0.001), the latter serving as an indicator of melanoma stage.

Consequently, we set out to test the differences in a multivariate scenario that included Pirin expression as our effect of interest, and age, sex and Breslow depth as possible co-variants. We further simplified our procedure by comparing the intense expression of Pirin (“High”) with the merged low levels of negative and low expression (“Low”). Stronger Pirin expression was significantly associated with an increased probability of metastasis as witnessed in a logistic regression model (*p* = 0.007, OR = 3.851, 95% CI [1.453, 10.213]: Fig. 1d). Furthermore, we performed a Bayes factors analysis to determine the probability of the alternative hypothesis, i.e.: the existence of an association between strong Pirin expression and metastasis. The result turned out to be positive, with a BF01 = 0.090 and a BF10 = 11.097, which implies that the existence of a relationship between strong Pirin expression and metastasis is 10 times more likely than the existence of no such effect. Finally, a Cox analysis showed that patients with stronger Pirin expression have more than twice the probability of developing early metastasis compared to those expressing Pirin weakly (*p* = 0.012, HR = 2.305, 95% CI [1.203, 4.417]), BF01 = 0.189, BF10 = 5.28: Fig. 1e).

### Pirin expression regulates the proliferation rates of melanoma cell lines

**Figure 2:**
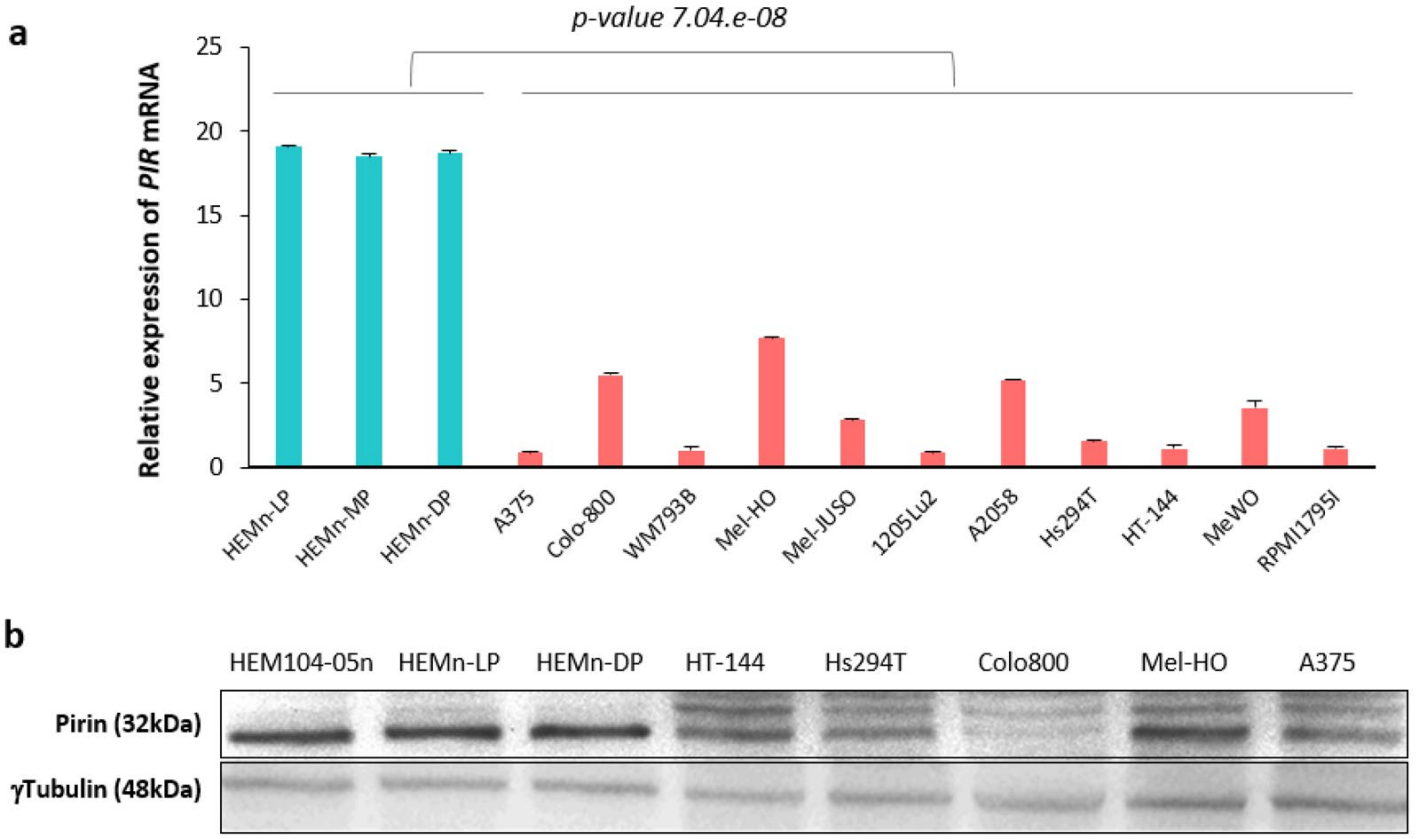
*PIR*/Pirin expression in cultured primary melanocytes and melanoma cell lines. (**a**) *PIR* mRNA expression in primary melanocytes and melanoma cell lines. RNA from three humans lightly (HEMn-LP), moderately (HEMn-MP), and darkly (HEMn-DP) pigmented neonatal foreskin melanocyte cell lines (blue), and from eleven melanoma cell lines (A375, Colo-800, WM793B, Mel-HO, Mel-JUSO, 1205Lu2, A2058, Hs294T, HT-144, MeWO and REPMI1795I: red), was analyzed by RT-qPCR. The average of three independent assays is shown. (**b**) Pirin protein in the four melanocyte cell lines and nine melanoma cell lines assessed in Western Blots, with Tubulin used as a loading control. The figure is representative of three independent cell culture experiments. Original blots are presented in Supplementary Fig. S1.

To further study the role of Pirin in the pathogenesis of melanoma, we evaluated the expression of Pirin in melanocytes and eleven melanoma cell lines by RT-qPCR and in Western Blots. The relative Pirin mRNA levels reflected the generalized weaker expression of the *PIR* gene in melanoma cell lines relative to the melanocytes from neonatal foreskin (*p* value = 7.04.e−08: Fig. 2a). Notably, heterogeneous mRNA levels were detected among the melanoma cell lines. Accordingly, when we analyzed the amounts of Pirin protein (Fig. 2b), less Pirin was evident in all the melanoma cell lines analyzed relative to the melanocytes.

These results were consistent with the generally stronger Pirin expression observed in benign nevi relative to the melanoma biopsies. The differences in Pirin expression observed between benign and malignant lesions, as well as in the cell lines, suggested a need to assess the biological processes regulated by Pirin in the context of melanoma. As such, MeWO and A2058 melanoma cells were transfected with a plasmid to overexpress *PIR*, and, essentially biological processes in cancer progression such as migration and cell proliferation were analyzed.

**Figure 3:**
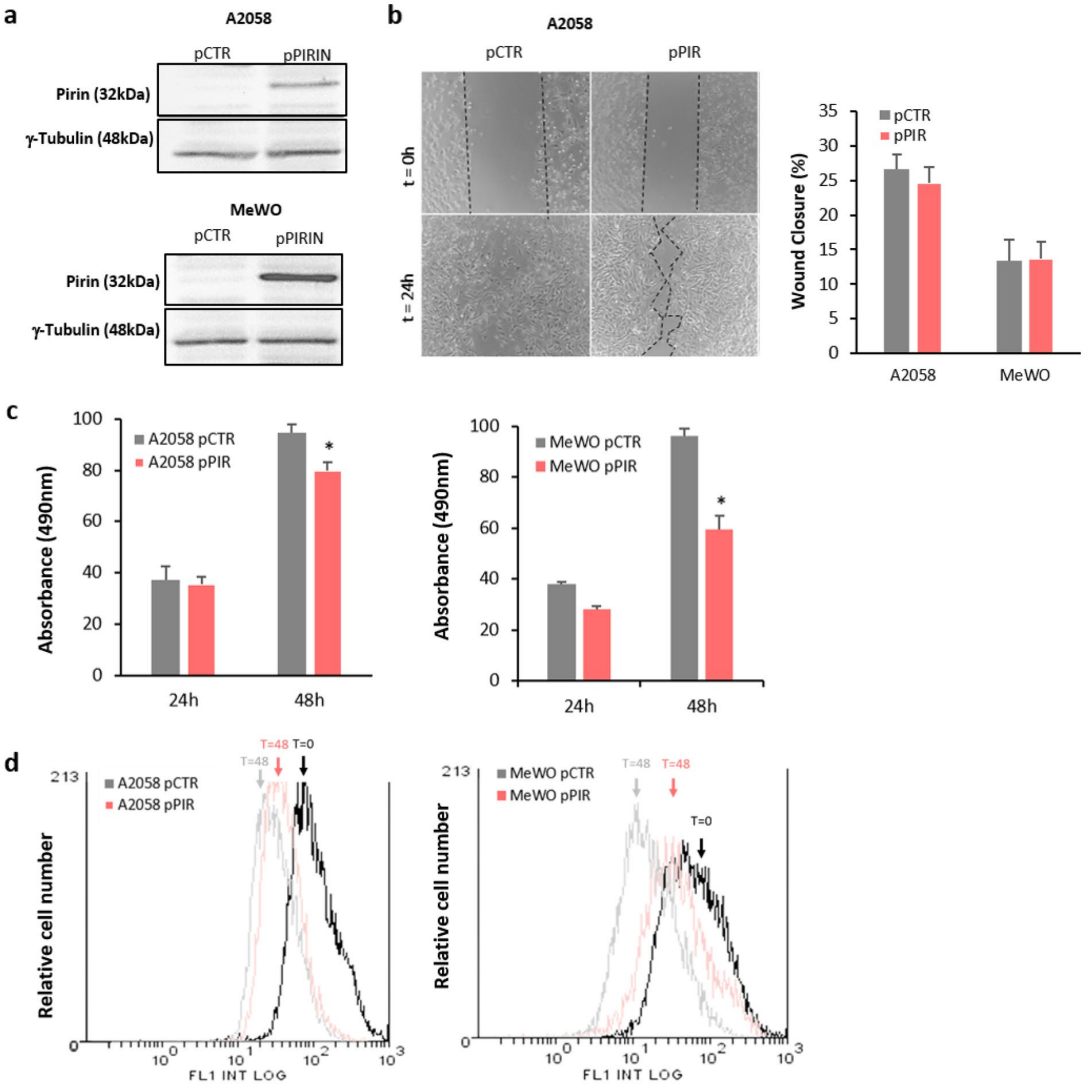
Functional assays after Pirin upregulation in melanoma cells. (**a**) Western Blot showing the Pirin upregulation in MeWO and A2058 melanoma cells, with Tubulin expression used as the loading control. Original blots are presented in Supplementary Fig. S2. (**b**) Change in the wound healing rate of A2058 and MeWO metastatic melanoma cells after Pirin upregulation. The experimental assays were performed at least 48 h after Pirin transfection. The histogram shows the average of three independent assays with six replicates per assay and representative images have been included. (**c**) The viability of control melanoma cells (transfected with an empty vector) and stable Pirin transfected clones was assessed in XTT assays over 24 and 48 h. The results of each experiment are expressed relative to the values obtained for the control transfections. The data are given as the mean ± SD of at least three independent experiments: *p value < 0.05. (**d**) The proliferation rate of A2058 and MeWO metastatic melanoma cells after Pirin upregulation was assessed by CFSE labeling over 48 h. The graphics show a representative histogram from three independent experiments.

Overexpression of PIR was verified by Western Blots, achieving a 5 to 15-fold increase in Pirin protein relative to the parental A2058 and MeWO melanoma cell lines, respectively (Fig. 3a, Supplementary file Fig. S2). When the wound-healing and proliferation of these cells was assessed, no significant differences were detected in the migration capacity between the control cells and those that overexpress Pirin (Fig. 3b) which was in line with the lack of consistent modifications in migration-sustaining EMT markers (Supplementary file, Fig. S3). By contrast, increased expression of Pirin led to a decrease in the viability of both cell lines, which was evident after 48 h (Fig. 3c). The reduction of viability was due to a decrease on the proliferation rate as determined by CFSE assay (Fig. 3d).

### Transcriptome modulation in healthy skin melanocytes after *PIR* down-regulation

**Figure 4:**
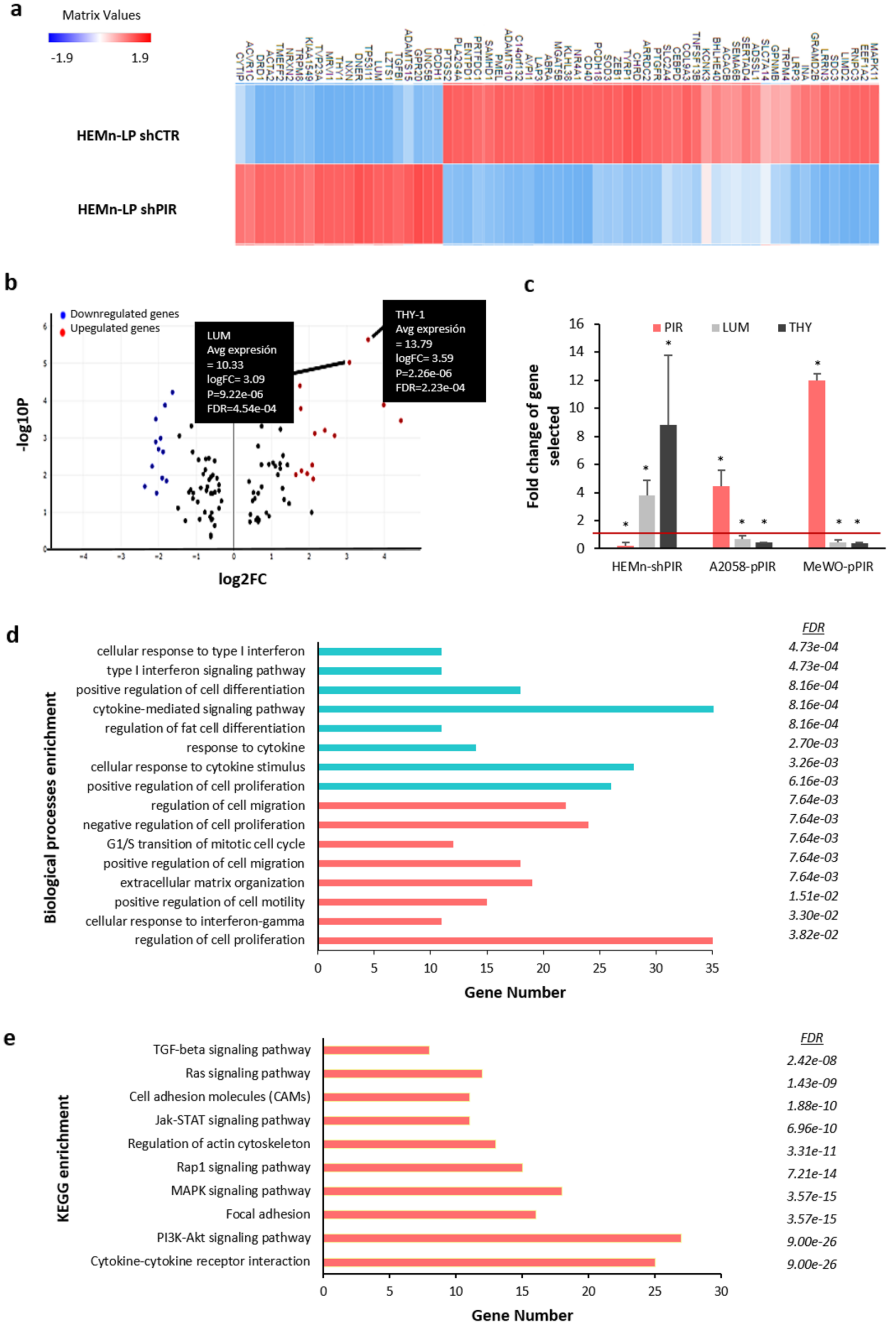
RNA Sequencing analysis and validation. (**a**) The clustergrammer displays the gene expression in for each sample in the RNA-seq dataset. Every row of the heatmap represents a sample and each column represents a gene. Prior to displaying the heatmap, the raw gene counts were normalized using the logCPM method, filtered by selecting the 100 genes with most variable expression and finally transformed using the Z-score method. (**b**) The figure contains a scatter plot which displays the log2-fold changes and statistical significance of each gene calculated by performing a differential gene expression analysis. Every point in the plot represents a gene. Red points indicate significantly up-regulated genes and blue points indicate down-regulated genes. Gene fold changes were transformed using log2 and displayed on the x axis; P-values were corrected using the Benjamini–Hochberg method, transformed using log10, and displayed on the y axis. (**c**) Validation of RNA Sequencing data using RT-qPCR o *PIR*, *LUM* and *THY-1* gene expression in HEMn-LP primary melanocytes with dampened Pirin expression, and in A2058 and MeWO metastatic cell lines transfected with a plasmid over-expressing Pirin. The average of three independent assays is shown and the red line highlights the normalized control expression: *p value < 0.05. (**d**) Bar charts displaying the results of the Gene Ontology enrichment analysis of Biological process generated using EnrichR. The x axis indicates the gene number for each term, the blue bars represent the terms enriched from the downregulated gene subset and the red bars represent the terms enriched from the upregulated genes subset. (**e**) Canonical pathways significantly modulated by *PIR* silencing. Every row of the figure represents one enriched process with an FDR cut-off of < 0.05, and each bar represents the number of genes included in each pathway.

Having assessed the capacity of Pirin to regulate basic tumor-related processes like proliferation, and in order to reveal the molecular mechanisms regulated by this protein, we performed a RNA-seq analysis. The strong Pirin expression observed in melanocytes and the lack of information regarding the role of Pirin in non-transformed cells led us to select this cell model to downregulate Pirin gene expression and to determine the transcriptomic alterations provoked. Pirin-specific shRNA expression induced a 90% reduction in *PIR* mRNA and a 30% loss of Pirin protein in HEMn-LP melanocytes (Supplementary file, Fig. S4a–b). Two independent replicates of control (shCTR) and *PIR* knockdown (shPIR) HEMn-LP samples were subjected to RNA sequencing. Initial quality control showed strong congruence of the biological replicates, with Spearman correlation values for all replicates of r = 0.81 (Supplementary file, Fig. S4c). This demonstrated the reliability of the data produced and illustrated transcriptional changes were consistent in each condition. A set of DEGs was identified when shCTR and shPIR HEMn-LP were compared based on standard threshold *p* value ≤ 0.05 and a FDR ≤ 0.05. In total, 824 DEGs were identified, of which 446 were downregulated and 374 were upregulated (Fig. 4a shows the genes with most variable expression after applying the logFC > (2) and logFC < (− 2) filter).

To validate the sequencing results, we focused on two genes linked to the malignant properties of melanoma cells, both of which underwent a significant increase in their mRNA levels in our dataset (Fig. 4b): Lumican (*LUM*), a gene related to growth and metastasis^20–22^; and THY-1, a cell surface antigen (*Thy-1/CD90*) that acts as an adhesion molecule for the extravasation of endothelial tissues and as a tumor suppressor^23,24^. We analyzed the expression of these two genes by RT-qPCR in HEMn-LP *PIR*-silenced cells, and in MeWO and A2058 metastatic melanoma cells overexpressing Pirin (Fig. 4c). The reduction in *PIR* mRNA was correlated with higher levels of *LUM* and *THY-1* mRNA. Conversely, overexpression of *PIR* mRNA was associated with weaker *LUM* and *THY-1* gene expression in transfected melanoma cells relative to the control cells. Together, *PIR* modulation observed appears to modify the transcriptome of cells, provoking the up and down-regulation of different genes.

To identify the biological processes that might be modified in response to *PIR* silencing, a functional enrichment analysis was performed on RNA sequencing datasets using the BioJupies Interactive Notebook, obtaining the Biological Processes (BPs) of that Gene Ontology (GO) analysis (Fig. 4d). Among the upregulated genes the BPs were mainly associated with extracellular matrix (ECM) organization, the regulation of migration, the regulation of proliferation and the cell cycle, and cell responses to type II interferon. For the downregulated genes, cell differentiation, cytokine-mediated signaling and innate immune response were the BPs predominantly altered. The GO annotation results (overlapping genes, *p* value and FDR) of the DEGs after *PIR* silencing are summarized in Table S1, and the top ten pathways in the pathway-enrichment analysis included: cytokine-cytokine receptor interaction, PI3K-AKT signaling pathway, focal adhesion, MAPK-signaling pathway, and JAK-STAT-signaling pathway (Fig. 4e). PI3K-AKT and cytokine-cytokine receptor interaction pathways were the most significantly enriched terms, both with an FDR = 9.00e−26.

### *JARID1B* a transcription factor putatively regulated by *PIR*

**Figure 5:**
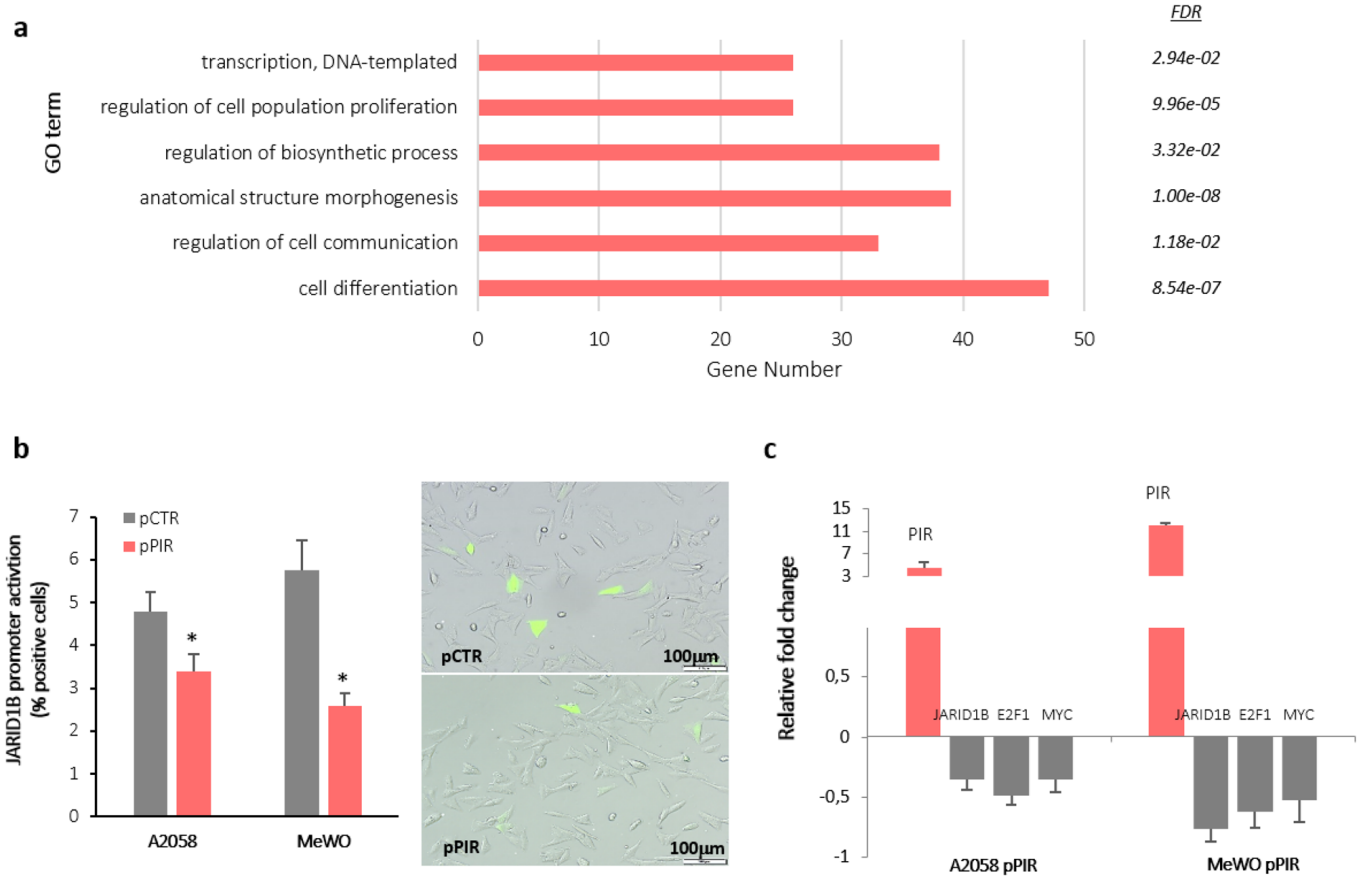
*PIR* as a key regulator of *JARID1B* expression in melanoma cells. (**a**) Gene ontology analysis of the subset of genes potentially targeted by *JARID1B*. Each row represents one enriched process, with a FDR cut-off of < 0.05, and each bar represents the number of genes associated with each pathway. (**b**) Cells positive for GFP from three independent pPIR|GFP-JARID1B co-transfection assays and a representative image from an A2058 cell assay: *p value < 0.05. (**c**) Expression of *JARID1B*, *E2F1* and *c-MYC* mRNA in *PIR* overexpressing melanoma cells by RT-PCR. The average of three independent assays is represented.

Due to the fact that Pirin acts as a transcriptional co-regulator with NF1 and NFkB^25^, we set out to determine whether Pirin modulated the expression of genes targeted by other transcription factors in melanocytes. A computational analysis of transcription factors likely to be associated with Pirin and its target genes was performed using the Biojupies Interactive Notebook, which significantly predicted that *JARID1B* could be one such transcriptional regulator working between *PIR* and its downstream modulated genes. *JARID1B* was recently described to be a slow cycling gene involved in the epigenetic regulation and malignant reprogramming of melanoma cells^26–28^. In our data, more than 100 DEGs were putative targets of this protein and significantly, the most strongly enriched GO terms for this subset of genes included regulatory processes related to transcription, proliferation, metabolism, morphogenesis, communication and differentiation (Fig. 5a and see a summary of the details of these GO entries in Table S2). It is also interesting to note that an association between *JARID1B* regulation and the PI3K-AKT pathway has been described, which was enriched in response to *PIR* silencing^29^. Therefore, we wondered whether *PIR* could somehow modulate *JARID1B* expression. To address this possibility, melanoma cells were co-transfected with a PIR-coding plasmid (pPIR) or an empty plasmid (pCTR) and a plasmid in which eGFP expression was driven by the *JARID1B* promoter. Activation of the *JARID1B* promoter was determined through the proportion of cells expressing GFP and significantly, an increase in Pirin expression in MeWO and A2058 melanoma cells led to a significant decrease in *JARID1B* promoter activation in both metastatic melanoma cell lines (Fig. 5b).

Based on our findings regarding the DEGs detected following PIR silencing in melanocytes, and the significant increase in *E2F1* (*p* value 5 × 10−4, FDR 0.022) and *c-MYC* (*p* value 7.8 × 10–5, FDR 0.006) expression observed, and given that these are target genes of *JARID1B*^30,31^, their expression was evaluated in Pirin overexpressing melanoma cells by RT-qPCR. Significantly weaker expression of the *JARID1B*, *E2F1* and *c-MYC* genes was clearly seen in melanoma cells that overexpressed Pirin relative to the control cells (Fig. 5c). Accordingly, it should be noted that the *THY-1* and *LUM* genes (whose expression is shown in Fig. 4c) are also targeted by *JARID1B*, and both genes are down-regulated in the metastatic melanoma cells overexpressing *PIR*. Moreover, the rates of proliferation assayed (Fig. 3c) were in agreement with this molecular analysis, since both *c-MYC* and *E2F1* are proteins involved in cell proliferation and cell cycle regulation. These data are consistent with the downregulation of *JARID1B* gene expression. Together, these results suggest that *PIR* might modulate melanoma proliferation by targeting the slow-cycling transcriptional regulator *JARID1B*.

## Discussion

Metastasis represents a significant problem in melanoma, even in early-stage (i.e. stage I–II) patients, since 10% of them relapse within five years after their initial diagnosis^32,33^. This is especially relevant for stage IIB and IIC patients (AJCC 8th edition) who often show worse disease progression than those diagnosed in stage III, in which sentinel node involvement, metastasis in transit or metastasis through microsatellites are detected^34^. In this context, efforts are now being directed towards the early characterization of patients, which is essential in a scenario where the absence of effective therapies for metastatic melanoma requires prognostic markers capable of discerning patients at greater risk of metastasis at early stages of the disease^34,35^. This search for early prognostic factors in melanoma is one of our main research objectives^36–40^, and here we focused on studying Pirin expression as an early diagnostic and prognostic marker of melanoma, as well as its role in the cellular mechanisms driving melanoma progression.

Pirin belongs to the superfamily of cupin proteins and it participates in the regulation of different cellular processes, acting as a protein kinase inhibitor, antioxidant or putative transcriptional co-factor^6,25,41–43^. Increased expression of Pirin has been associated with lymph node metastasis in breast cancer^44^ and enhanced expression of this protein has been also observed in colorectal tumors^17^. In melanocyte context, a homogenously high Pirin expression in benign melanocytes from nevi was observed, in contrast to the heterogeneous expression in malignant melanomas. Focusing on stage I and II melanoma patients, we explored whether Pirin expression might be related to a more aggressive phenotype. The patients were divided into disease-free patients and patients who developed metastasis during follow-up. Based on multivariate analyses by Logistic Regression and Cox models, including age and the Breslow index as co-variates, strong Pirin expression was significantly associated with a risk of metastasis, suggesting its importance as a prognostic marker.

Regarding the functional role of Pirin, this protein has been shown to promote cell proliferation and migration in breast cancer models^45^ and to support cell migration favoring EMT process in cervix carcinoma cells^14^. Moreover, inhibition or decrease of Pirin in melanoma has been shown to diminish cellular migration capacity of melanoma cells^13,18,25^. Nevertheless, neither proliferation nor migration were affected in colon carcinoma cells^17^. We studied the proliferation and migration of metastatic melanoma cells in which Pirin was overexpressed and we found this upregulation did not modify migration but rather, it did induce a significant decrease in the proliferation rate of both the melanoma cell lines studied. Discrepancies among results may be linked to the tumor type or origin as role of Pirin in EMT-linked increase on migration capacity has been observed in tumors of epithelial origin while melanoma arises from the malignant transformation of neural crest-derived non-epithelial cells^46^. In fact, in our settings, Pirin overexpression did not lead to an alteration on Vimentin or N-Cadherin levels. Lack of effect on migration may also be linked to the methodological approach as migration has been always being affected by the decrease or inhibition of Pirin instead of overexpression-based settings^14,16^. In addition, Pirin has been shown to interact with NF-1, BCL-3 and NF-kB, forming an activated complex able to promote previously mentioned EMT and cell migration^13^. Here, BCL-3 expression in nevi and melanoma biopsies was analyzed in parallel to Pirin expression, yet no relationship between them was found (Supplementary file, Fig. S5). Hence, other cellular signaling pathways would appear to be involved in the metastatic activity of Pirin in melanoma cells. On the other hand, the pro-proliferative role of Pirin has been based on its capacity to support E2F1 expression^45^ while our results, in both melanoma cell lines, indicate the opposite effect suggesting a cell-type dependent effect.

The cellular activity of Pirin has mainly been studied in terms of extracellular matrix tumorigenicity^16,25,47^ and thus, despite its broad distribution, there is little information regarding the role of Pirin in non-transformed cells and tissues^6,48^. To better understand the role of Pirin in a melanocytic context, we firstly studied Pirin expression in primary melanocytes and melanoma cell lines by RT-qPCR and in Western Blots, demonstrating that primary melanocytes exhibit generally stronger and more homogenous Pirin expression than melanoma cell lines, which had significantly lower expression and more heterogeneity among the different cell lines analyzed.

In accordance with the antiproliferative activity observed when Pirin is overexpressed in melanoma cells, the transcriptomic analysis following *PIR*-silencing in primary melanocytes here revealed an enrichment of genes involved in the negative regulation of cell proliferation, the G1/S transition and extracellular matrix organization and positive regulation of cell migration (Table S1). Furthermore, deleterious mutations in the *PIR* gene were recently identified in breast cancers that could affect protein structure, stability and function^45^. These results could explain the discrepancies found when studying different cancers or different tumor cell lines. On the other hand, melanoma heterogeneity was recently proposed to be due to the co-existence of different melanoma cell phenotypes and adaptive phenotype plasticity given that transcriptional reprogramming could drive melanoma progression^49^. Transcriptional reprogramming has been detected at different stages of melanoma, with enhanced mesenchymal traits in circulating melanoma cells and proliferative features in metastatic tumors^49^. Hence, cells with different phenotypes may interact in a cooperative manner and contribute to successful metastatic progression^50,51^.

In recent years, attention is paid to epigenetic regulation in melanoma^28^, which led to the description of *JARID1B* as an epigenetic regulator implicated in the transcriptional reprogramming of several tumor cells and in tumor heterogeneity^52^. Although JARID1B expressing melanoma cells represent only a small proportion of the cells in the primary and metastatic melanoma populations^53^, the RNA-seq dataset and the transcription factor enrichment analysis found that *JARID1B* could target more than 100 of the DEGs identified. Furthermore, co-transfection experiments showed a decrease of *JARID1B* promoter activation after Pirin overexpression, pointing to a functional relationship between Pirin and JARID1B expression. In addition, we demonstrated that the overexpression of Pirin in both the metastatic melanoma cell lines studied led to a significant decrease in *JARID1B* gene expression, and that of its target genes *E2F1* and *c-MYC*^30,31^. These results may explain the antiproliferative effect of Pirin observed in melanoma cell lines. Indeed, in canine oral melanoma cell lines JARID1-inhibitors drove anti-proliferative activity and overcame cisplatin resistance^54^.

From our data, we believe that in melanocytes Pirin expression could regulate the rate of proliferation through *JARID1B* and *E2F1* pathway^39,55–57^. Moreover, as JARID1B is involved in the neural differentiation process^58^, we hypothesize that it could be maintaining the undifferentiation state of the low proliferative Pirin expressing melanoma cells and potentially a more aggressive phenotype. Indeed, tumor cells with a slow-cycling phenotype may be metabolically active and highly aggressive, with increased potential to grow and metastasize^27,59,60^.

In summary, our study supports the use of Pirin staining along with the Breslow index as a prognostic marker at early stages (I–II) of melanoma. High Pirin protein levels are associated with a more significant probability of metastasis, as well as a shorter time until this clinical end-point. In addition, we propose that Pirin could play an important role in modulating the differentiation and proliferative state of melanoma cells by regulating *JARID1B* gene expression. However, further research will be necessary to better understand the mechanisms underlying this phenomenon, which could shed light on useful therapeutic strategies for these tumors.

## Methods

### Patients

A total of 314 patients (75 nevi and 239 melanomas) were recruited between 1990 and 2016 at the Dermatology Units of the Basurto and Cruces University Hospitals. The inclusion criteria were: (1) a histologically confirmed diagnosis of nevus or malignant melanoma; (2) no treatment except primary surgery; (3) no infection as judged by clinical evaluation and the absence of any increase in parameters related to infections in the blood.

After surgery of the primary tumor, clinical check-ups were scheduled every three months for the first two years of the follow-up, and every six months thereafter, until a five-year follow-up had been completed. Annual revisions were then scheduled up to the tenth year post-surgery. The patients who developed metastasis during the follow-up period were again examined every three months for two years after their metastasis had been diagnosed. The presence or absence of metastasis was assessed in all patients by physical examination, as well as through laboratory and radiological testing (X-rays and/or computed tomography -CT- scanning). Metastases were detected in 92 of the 239 melanoma patients studied (38%), including those in whom the disease had already spread at the moment of diagnosis. Disease stages were classified according to the AJCC 8th edition^19^, and the clinical and diagnostic data for each patient was collected retrospectively from centralized electronic and/or paper medical records. For the statistical prediction analysis, only melanoma patients at early disease stages (I and II) were considered (159 subjects in total), and inclusion in the “disease-free” group required a minimum tracking of 2 years.

The study was carried out in accordance with the Helsinki Declaration and it was approved by the Drug Research Ethics Committee of the Department of Health of the Basque Government (reference 16–99) (http://www.euskadi.eus/comite-etico-investigacion-con-medicamentos/). Written informed consent was obtained from all the subjects and the melanoma biopsies collected were stored at the Basque Biobank until use (https://www.biobancovasco.org/). For more details about the informed consent see *supplementary material*.

### Immunohistochemistry

Sections (4 μm thick) from Formaldehyde Fixed Paraffin Embedded (FFPE) blocks were subjected to antigen retrieval in citrate buffer (pH 6.1) and steam for 105 min, and then analyzed by Pirin immunohistochemistry with an anti-Pirin antibody (PA5-29777: Thermo Fisher Scientific, Waltham, MA, USA) and using the EnvisionTMG|2 Sistema/AP Kit (Dako Corporation, Denmark). The slides were counterstained with hematoxylin and images were obtained using a NanoZoomer S210 Digital slide scanner (Hamamatsu C13239-01). The staining intensity was evaluated as negative, low or high expression after independent examination by two observers. Discordant assessments were reviewed jointly to obtain a conclusive consensus evaluation.

### Statistical analysis

Bivariate statistical testing of categorical parameters (e.g. sex and melanoma prognosis) was performed with Pearson’s chi-squared tests, whereas the Cochran-Armitage Test was employed when Pirin was involved. Statistical differences between two or more groups were estimated with a two-tailed Kruskal-Wallis test or by ANOVA if normality was assumed based on the Shapiro-Wilk test. A logistic regression model was employed to test the association between Pirin and prognosis, adjusting for possible sources of co-variation. For this analysis, marginal effects were also computed^61^. Finally, a Cox Proportional-Hazard model was established to test the association of Pirin expression with the onset of metastasis (measured in months). All p-values were computed non-parametrically using the R package “coin”^62^, controlling for the false discovery rate (FDR) in the case of multiple testing^63^, and statistical evidence of the effects was quantified using the BIC (Bayesian Information Criterion) and Bayes factors^64^. The Bayes Factors indicate the probability of the alternative hypothesis (in our case, there is an association of Pirin expression with metastasis) relative to the null hypothesis (no association of Pirin expression with metastasis) and vice versa, such that: BF01 = Probability (Null Hypothesis)/Probability (Alternative Hypothesis); and BF10 = 1/BF01 (the inverse). All the analyses were carried out using R version 3.6.1.

### Cell lines, and the proliferation and migration assays

In the present work, eleven melanoma cell lines and three primary melanocyte cell lines were studied. The primary human melanocytes were purchased from Invitrogen (Carlsbad, CA, USA), and they correspond to lightly (HEMn-LP, C-002-5C), moderately (HEMn-MP, C-102-5C) and darkly (HEMn-DP, C-202-5C) pigmented neonatal foreskin. All primary human melanocytes were grown in Cascade 254 Medium supplemented with Cascade Human Melanocyte Growth Supplement (Invitrogen; Carlsbad, CA, USA) in the absence of antibiotics. Some melanoma cell lines were purchased from the American Type Culture Collection (Rockville, MD, USA): A375 (ATCC CRL-1619), A2058 (ATCC CRL-11147), Hs294T (ATCC HTB-140), HT-144 (ATCC HTB-63), MeWo (ATCC HTB-65), WM793B (ATCC CRL-2806), and 1205Lu (ATCC CRL-2812). The remainder were obtained from Innoprot (Derio, Bizkaia, Spain): RPMI7951 (ACC76), COLO-800 (ACC193), MEL-HO (ACC62), and MEL-Juso (ACC74). The melanoma cell lines were cultured in an appropriate medium supplemented with 10% fetal bovine serum (FBS), L-glutamine (2 mM) and antibiotics, according to the supplier’s indications. All the primary human melanocytes and melanoma cell lines were cultured at 37 °C in and atmosphere of 5% CO_2_ and 95% humidity.

For monitoring cell proliferation, the control melanoma cells and the melanoma cells with stable PIR gene transfected clones were labeled with CFSE, a dye which is retained in the viable cells and gradually halves as the cells division. Briefly, desired density grown cells were incubated in pre-warmed PBS containing CFSE working solution (1 μM). After incubation, cells were detached and fix in paraformaldehyde 4% and analyzed by flow cytometry. The reduction in CFSE fluorescence signal was visualized by computer analysis using WEASEL 3.7.1—Flow Cytometry Data Analysis software. Moreover, we carried out the 2,3-bis-(2-methoxy-4-nitro-5-sulfophenyl)-2H-tetrazolium-5-carboxanilide (XTT) assay, following the manufacturer’s instructions. Absorbance at 490 nm was measured at 24 and 48 h, and cell viability was calculated in relation to untreated control cells as: (experimental absorbance/untreated control absorbance) × 100.

In migration assays, after seeding in 24-well plates the cell monolayers were incubated with Mitomycin C (0.5 µg/mL) for 2 hours and they were then scraped with a sterile plastic micropipette tip. Wound closure was observed over 48 h, taking photos at 0, 24 and 48 h under a light microscope.

### Transfection and transduction assays

Normal primary human melanocytes HeMn-LP were transduced with PIR shRNA Lentiviral Particles (sc-61359-V) or Control shRNA Lentiviral Particles (sc-108080: Santa Cruz Biotechnology Inc., Dallas, TX, USA) following the manufacturer’s instructions. Two days later the transduced cells were selected with Puromycin (P8833, 5 µg/mL: Sigma Aldrich, San Luis, MO, USA) to obtain stable cell lines. A2058 and MeWO metastatic melanoma cell lines were transfected using Lipofectamine 2000 (Thermo Fisher Scientific, Waltham, MA, USA) with a plasmid to overexpress *PIR* (pCMV-PIR-myc-DKK: OriGene Technologies, Inc, Rockville, MD, USA) or the empty plasmid, following the manufacturer’s instructions. All of the transfection experiments were performed with 500 ng of DNA and the experimental assays were performed at least 48 h post-transfection.

To study the activity of the *JARID1B* gene induced by Pirin, the *JARID1B* promoter was cloned into a plasmid vector containing the coding region of the enhanced green fluorescence protein (eGFP gene, EWI024-FP pLU-JARID1B1B promoter-EGFP Lentiviral Plasmid: Kerafast, Boston, MA, USA). This plasmid was co-transfected with the plasmid to overexpress *PIR* in the A2058 and MeWO metastatic melanoma cell lines. At 48 hours post-transfection, cells with positive GFP fluorescence were visualized by fluorescence microscopy (Zeiss, OberKochen, Germany).

### Western blotting

Melanoma cells and primary melanocytes were harvested by trypsinization, washed with phosphate buffer saline (PBS), and lysed in RIPA lysis buffer (80 mM Tris-HCl [pH 8], 150 mM NaCl, 1% NP 40, 0.5% sodium deoxycholate, 0.1% Sodium Dodecyl Sulfate -SDS) containing a Protease Inhibitor Cocktail (Sigma-Aldrich Quimica S.A., Madrid, Spain). After 15 minutes on ice in lysis buffer, the lysates were cleared by centrifugation at 10,000 *g* for 5 min and the concentration of the total protein recovered was determined using the bicinchoninic acid assay.

For Pirin protein detection, total protein (40 µg) from each sample was resolved by electrophoresis on SDS-polyacrylamide gels and then transferred to a nitrocellulose membrane (Whatman GmbH, Dassel, Germany). The membranes were incubated with PBS containing 5% Bovine Serum Albumin (BSA) and 0.1% Tween-20 for 1 hour to block non-specific binding, and they were then probed overnight at 4 °C with an appropriate dilution of the primary antibodies against Pirin (1:1,000, PA5-29777: Life Technology, Carlsbad, CA, USA) and γTubulin (1:3000, #T9026: Sigma-Aldrich, San Luis, MO, USA). The membranes were then washed three times (10 min/each) with TBST (Tris-buffered saline with Tween 20), then incubated for 2 h at room temperature with a goat anti-mouse Horseradish Peroxidase (HRP) conjugated secondary antibody (1:10,000, anti-mouse #1032-05: Southern Biotechnology, Birmingham, AL, USA). Finally, antibody binding was visualized by enhanced chemiluminescence (ECL) using the SuperSignal^®^ West Pico Chemiluminescent Substrate (Thermo Scientific, Rockford, IL, USA).

### Sequencing analysis

For RNA Sequencing, the RNA concentration was first measured in a Qubit 2.0 RNA assay Kit (Invitrogen, Carlsbad, CA, USA, #Q32855). The quality of all the RNAs assayed was characterized on an Agilent 2100 Bioanalyser using an Agilent 6000 Nano Chip #5067-1511, with an optimal RNA Integrity Number (RIN) between 8 and 10. Sequencing libraries were prepared following the “TruSeq Stranded mRNA Sample Preparation Guide (Part # 15031058 Rev. E)” with the corresponding kit [Illumina Inc., #RS‐122‐2101 or RS‐122‐2102 (Set A or B, respectively)]. Briefly, mRNA was purified from the total RNA (400 ng), fragmented and primed for cDNA synthesis. The first strand was synthesized for 10 min at 25 °C, 15 min at 42 °C and 15 min at 70 °C using the SuperScript‐II Reverse Transcriptase (Thermo Fisher Scientific, #18064‐014), and then holding the reaction at 4 °C. The second cDNA strand was synthesized with Illumina reagents at 16 °C for 1 hour before A‐tailing and adaptor ligation was performed. Finally, library enrichment was achieved by PCR (30 s at 98 °C, 15 cycles of 10 s at 98 °C, 30 s at 60 °C, 30 s at 72 °C, 5 min at 72 °C and holding at 4 °C). The libraries were then visualized on an Agilent 2100 Bioanalyser using Agilent High Sensitivity DNA kit (Agilent Technologies, #5067‐4626) and quantified using Qubit dsDNA HS DNA Kit (Thermo Fisher Scientific, Waltham, MA, USA Scientific, #Q32854). Single-end sequencing was performed on the Illumina platform HiSeq2500, resulting in 51 bp reads after discarding the final base. The images taken during the sequencing reactions were processed using Illumina’s sequencing control software and base calling was achieved through the RTA software. The Platform for Genome Analyses at the Center for Cooperative Research in Biosciences (CIC bioGUNE—member of the Basque Research and Technology Alliance) was responsible for carrying out both the library and sequencing steps.

The raw data have been deposited in the NCBI Sequence Read Archive (SRA) through the Gene Expression Omnibus (BioProject accession number PRJNA843921). The reads were initially mapped to the reference genome from the UCSC genome browser (Human GRCh38/hg38 version) using Hisat2. The reads were assembled into transcripts using StringTie and the transcript read count was quantified using the feature counts software of the Subread package in the R statistical environment. Differentially expressed genes (DEGs) were identified by means of the edgeR package in R-studio, implementing a negative binomial distribution to assess statistical significance. Normalization was performed using the trimmed mean of M values (TMM) method. For each gene, the reads per kilobase of exon model (RPKM) per million mapped reads were transformed to log2-fold changes using R version 3.1.0. The analysis of variance (*p* < 0.05) and false discovery rate (FDR < 0.05) tests were performed using the R program (version 3.1.0) to select genes exhibiting significantly different expression patterns. The upregulated and downregulated DEG sets were generated by extracting the 500 genes with the respectively highest and lowest values from the gene expression signature. Spearman’s rank correlation coefficient analysis (SRCCA) was used to determine the strength of association between samples. Enrichment results were generated by analyzing the upregulated and downregulated gene sets using BioJupies^65^ and EnrichR^66^, which are freely available at https://amp.pharm.mssm.edu/biojupies and http://amp.pharm.mssm.edu/Enrichr, respectively. A KEGG pathway enrichment analysis of the DEGs was performed using the KOBAS online database (available online: http://kobas.cbi.pku.edu.cn/).

### RT-qPCR analysis

Total RNA was isolated from the cultured cells using the RNeasy Mini kit (Qiagen Inc, Hilden, Germany) and cDNA was synthesized from the total RNA (1 µg) from each sample using the iScriptTM cDNA Synthesis kit (Bio-Rad, Hercules, CA, USA), according to the manufacturer’s instruction. Quantitative real-time RT-PCR (RT-qPCR) assays were carried out on an iCycler PCR platform (Bio-Rad, Hercules, CA, USA). The reaction mixture contained cDNA (0.1 µl) from the reverse transcription reaction, together with forward and reverse specific primers (see Table 2), and the iQTM SYBR^®^ Green Supermix (Bio-Rad, Hercules, CA, USA) in a final reaction volume of 20 µl.

**Table 2:** RT-qPCR primer sequences.

Gene name	Forward primer	Reverse primer
*PIR*	5′-GGAGCCTCAGTACCAGGAACT-3′	5′-CTTGGACTTTATTCCCAGGGC-3′
*LUM*	5′-AACTGCCCTGAAAGCTACCC-3′	5′-AGCCACTGCAGATCAGTTACA-3′
*THY-1*	5′-GTTTGACCAGGAAAGCAGCG-3′	5′CTCTTGGGAGCTTGGGACAG-3′
*JARID1B*	5′-GACTGGGACAACAGAACCT-3′	5′-TGGACTAACACCATGGAGG-3′
*E2F1*	5′-TGACATCACCAACGTCCTTGA-3′	5′-CTGTCGGAGGTCCTGGGTC-3′
*c-MYC*	5′-GCTCCTGGCAAAAGGTCAG-3′	5′-GTTGTGCTGATGTGTGGAGAC-3′
*ACTB*	5′-AGATGACCCAGATCATGTTTGAG-3′	5′-GTCACCGGAGTCCATCACG-3′
*GAPDH*	5′-CCTGTTCGACAGTCAGCCG-3′	5′-CGACCAAATCCGTTGACTCC-3′
*RPS15*	5′-CGACCAAATCCGTTGACTCC-3′	5′-CGGGCCGGCCATGCTTTACG-3′

The PCR began by heating the reaction at 95 °C for 10 min, followed by 45 cycles of denaturation at 95 °C for 30 s, annealing at the corresponding temperature for each gene (56–61 °C) for 20 s and extension at 72 °C for 30 s. Each assay included a negative control (a sample without cDNA). The expression data were generated from two amplification reactions with the samples and controls run in triplicate. Optical data obtained by RT-PCR were analyzed using the MyiQ Single-Color Real-Time PCR Detection System Software v.1.0 (Bio-Rad). Analysis of the melting curve for each PCR assay and 1.5% agarose gel electrophoresis analysis of randomly selected samples was performed to confirm the specificity of the amplification products. The expression of three different housekeeping genes (ACTB, GAPDH and RPS15) was also analyzed to normalize the expression data using the Gene Expression Macro Software Version 1.1 (Bio-Rad Laboratories, Hercules), where the relative expression values were calculated by the comparative Ct method^67,68^.

## Supplementary Information

Supplementary Information.

## Data Availability

The datasets generated and analyzed in the current study are available at the BioProject repository with the accession number PRJNA843921 (https://www.ncbi.nlm.nih.gov/bioproject/PRJNA843921).
