# Supplementary material for: Pirin is a prognostic marker of human melanoma that dampens the proliferation of malignant cells by downregulating JARID1B/KDM5B expression
Source: Sci Rep. 2023 Jun 12;13:9561. doi: 10.1038/s41598-023-36684-2 (PMC10260996; doi:10.1038/s41598-023-36684-2)
Supplement: Supplementary file 1 — Supplementary Information. [file 41598_2023_36684_MOESM1_ESM.docx]

**Pirin is a prognostic marker of human melanoma that dampens the proliferation of malignant cells by downregulating *JARID1B/KDM5B* expression**

# **Cristina Penas^1^, Yoana Arroyo-Berdugo^1^, Aintzane Apraiz^1,2^, Javier Rasero^3^, Iraia Muñoa-Hoyos^4^, Noelia Andollo^1,2^, Goikoane Cancho-Galán^5^, Rosa Izu^2,6^, Jesús Gardeazabal^2,7^, Pilar A. Ezkurra^1^, Nerea Subiran^2,4^, Carmen Alvarez-Dominguez^8^, Santos Alonso^2,9^, Anja K. Bosserhoff^10,11^, Aintzane Asumendi^1,2^ and María D. Boyano^1,2*^**

**
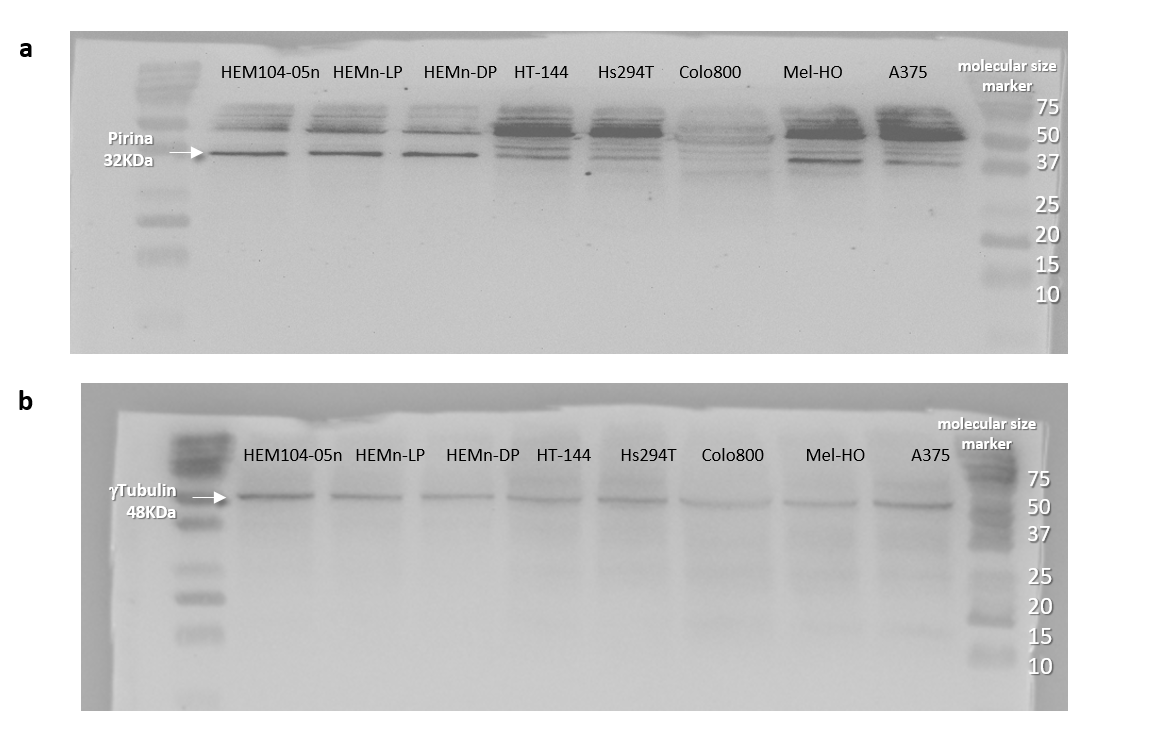
**

**Figure S1**. Western blots of melanocyte and melanoma cell lines. Details of the blots from Figure 2 showing. **a.** Pirin (32Kda) and **b.** γTubulin (48kDa) expression in human epidermal melanocytes and melanoma cell lines.

**
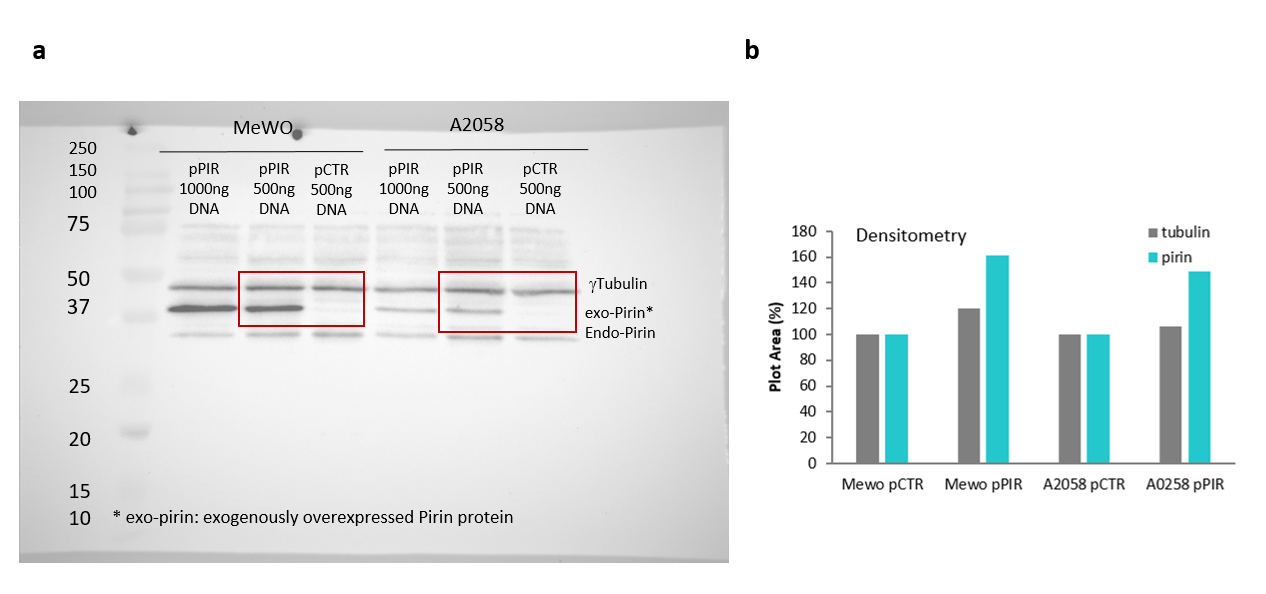
**

**Figure S2**. Western blots of Pirin-overexpressing melanoma cell lines. **a.** Details of the blots from Figure 3 showing Pirin expression in overexpressing MeWO and A2058 melanoma cell lines. Pirin plasmid (Myc-DDK-tagged) encodes for a Pirin protein 4KDa bigger than the endogenous Pirin. Therefore, blots belonging to transfected samples contain 2 lines (endogenous and overexpressed forms of Pirin). Red squares highlight the blot fraction employed for the Figure 3 (horizontal rotation). **b** Densitometry of the blots showing the relative levels of Pirin and γ-tubulin.

***
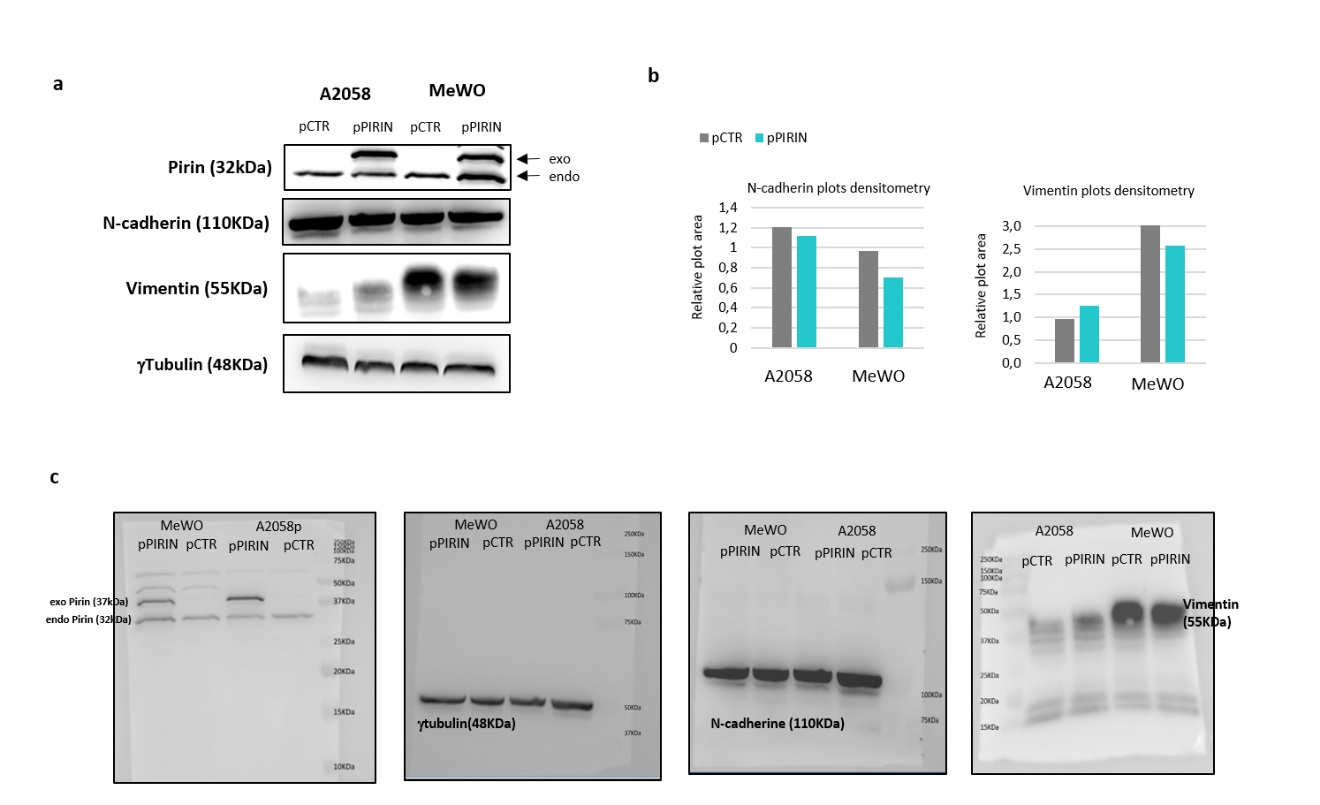
***

**Figure S3. a**. Western Blot showing the mesenchymal markers, n-cadherin and, vimentin after pirin overexpression. γTubulin expression was used as the loading control. **b** Densitometry of plots area of n-cadherin and vimentin relative to γTubulin amount. **c**. Details of the original blots.


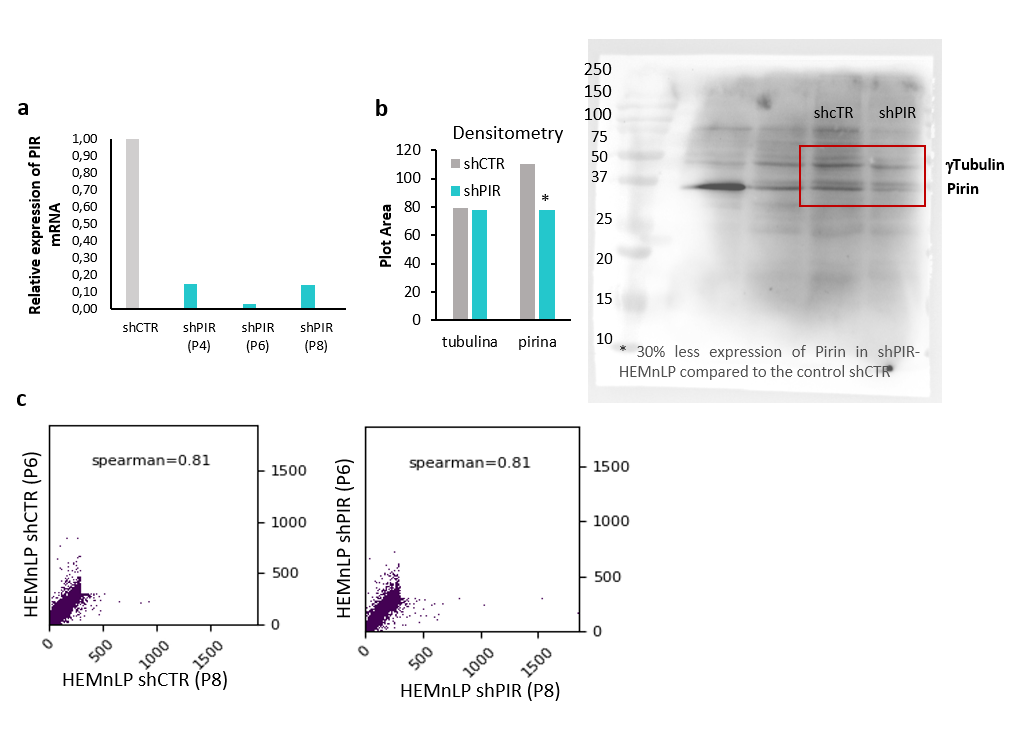


**Figure S4. Silencing of *PIR* in HEMn-LP cells. a.** Relative expression of *PIR* mRNA in three consecutive passages of primary melanocytes (HEMn-LP) after Pirin downregulation. ACTB was used as a housekeeping gene for relative quantification. The average of three independent assays are shown**. b.** Original blot of protein extracts from primary HEMn-LP melanocytes (line 1-CTR), control of transfection efficiency (line 2-GFP), empty plasmid (line 3-shCTR), *PIR*-silenced (line 4-shPIR) and densitometry showing the relative level of Pirin/γtubulin. Red squares highlight the blot fraction employed for the densitometry quantification. **c.** Spearman correlation coefficient between replicates of *PIR*-silenced HEMn-LP cells.


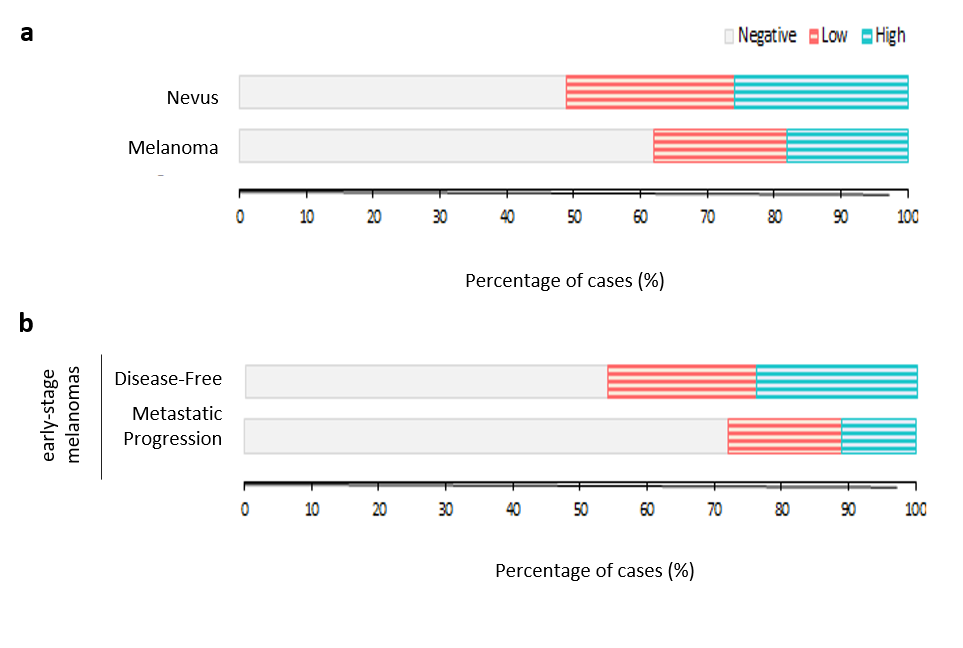


**Figure S5. BCL3 protein expression in melanoma biopsies. a.** Comparative analysis of the percentage of cases for each level of BCL3 expression between nevi and melanoma. **b**. Early-stage melanoma (I+II stages) divided by patients who remained disease-free or patients who developed metastasis during the follow-up.

**Table S1.** Enriched processes after PIR silencing, specifying the False Discovery Rate for assignment and the list of genes for each one.

| **Enriched term** | **FDR** | **Overlapping genes** |
| --- | --- | --- |
| regulation of cell proliferation | 3,82E-02 | ['CXCL6', 'BNC1', 'PDGFB', 'PDGFA', 'FGF1', 'TOB1', 'ADGRG1', 'GPNMB', 'MYC', 'PDGFD', 'GPER1', 'NTF3', 'ZNF503', 'SPEG', 'TP53I11', 'TRPM4', 'CGREF1', 'JUN', 'JUP', 'IGFBP3', 'TNK2', 'DAB2IP', 'P3H2', 'SOX11', 'CDC6', 'INHBA', 'NGF', 'PODN', 'SHCBP1', 'AGT', 'FOSL1', 'DDAH1', 'PRC1', 'BHLHE40', 'CDH13'] |
| cellular response to interferon-gamma | 3,30E-02 | ['CCL13', 'CIITA', 'CCL8', 'OAS1', 'CCL7', 'IRF7', 'HLA-F', 'TRIM21', 'HLA-DQA1', 'OASL', 'HLA-DQB1'] |
| positive regulation of cell motility | 1,51E-02 | ['SEMA7A', 'WNT5B', 'PDGFB', 'PDGFA', 'FGF1', 'MYLK', 'CCL7', 'GPNMB', 'PDGFD', 'GPER1', 'NTF3', 'SNAI1', 'CDH13', 'DRD1', 'GTSE1'] |
| extracellular matrix organization | 7,64E-03 | ['COL13A1', 'LUM', 'PDGFB', 'TNC', 'PDGFA', 'ADAM19', 'ACAN', 'MMP16', 'COL4A2', 'COL4A1', 'COL8A1', 'COL10A1', 'TGFBI', 'A2M', 'GAS6', 'ADAMTS9', 'RECK', 'JAM3', 'ITGA9'] |
| positive regulation of cell migration | 7,64E-03 | ['TNFSF18', 'SEMA7A', 'WNT5B', 'PDGFB', 'DAB2IP', 'PDGFA', 'FGF1', 'MYLK', 'GPNMB', 'CCL7', 'PDGFD', 'GPER1', 'NTF3', 'SNAI1', 'ENPP2', 'CDH13', 'DRD1', 'GTSE1'] |
| G1/S transition of mitotic cell cycle | 7,64E-03 | ['DHFR', 'POLA2', 'RRM2', 'MYC', 'PLK2', 'EIF4EBP1', 'E2F1', 'RCC1', 'MCM4', 'INHBA', 'CDC6', 'BCAT1'] |
| negative regulation of cell proliferation | 7,64E-03 | ['CGREF1', 'IGFBP3', 'P3H2', 'DAB2IP', 'SOX11', 'CDC6', 'INHBA', 'PODN', 'NGF', 'TOB1', 'ADGRG1', 'ACVR1C', 'GPNMB', 'DDAH1', 'MYC', 'GPER1', 'BHLHE40', 'E2F1', 'SPEG', 'CDH13', 'ZNF503', 'IL12A', 'TRIB1', 'TP53I11'] |
| regulation of cell migration | 7,64E-03 | ['SEMA7A', 'WNT5B', 'PDGFB', 'PDGFA', 'NEXN', 'THY1', 'PODN', 'FGF1', 'MYLK', 'TMEFF2', 'GPNMB', 'CCL7', 'PDGFD', 'GPER1', 'NTF3', 'SNAI1', 'ENPP2', 'CDH13', 'DRD1', 'AMOTL1', 'GTSE1', 'RECK'] |
| positive regulation of cell proliferation | 6,16E-03 | ['PTGFR', 'CD81', 'CHRD', 'LRP5', 'TNFSF13B', 'DPP4', 'PDGFD', 'GPER1', 'S1PR3', 'IL6R', 'TRPM4', 'NRG1', 'AGT', 'PGF', 'ADRA2A', 'TGFBR2', 'FOSL1', 'BMP4', 'TGFBR3', 'BMP2', 'IL6', 'SFRP2', 'IL7', 'RARA', 'HPSE', 'GAS6'] |
| cellular response to cytokine stimulus | 3,26E-03 | ['CCL13', 'CEBPD', 'LRRC3', 'PTGS2', 'IFIT1', 'ICAM1', 'CCL8', 'CCL7', 'FLRT3', 'GPER1', 'DPYSL3', 'ZC3H12A', 'IL12A', 'CCR7', 'JUNB', 'IL6R', 'IFNLR1', 'IRAK3', 'BATF', 'MAPK11', 'IL6', 'ZEB1', 'RTN4RL1', 'IL7', 'IRF4', 'BCL2', 'RHOU', 'GAS6'] |
| response to cytokine | 2,70E-03 | ['GCH1', 'MX1', 'IFNLR1', 'IRAK3', 'PLSCR1', 'KYNU', 'DPYSL3', 'BCL2', 'CCR7', 'JUNB', 'IL6R', 'TRIM21', 'TIMP4', 'SNCA'] |
| regulation of fat cell differentiation | 8,16E-04 | ['BMP2', 'IL6', 'SFRP2', 'FRZB', 'GPER1', 'LEP', 'ZC3H12A', 'LRP5', 'ZNF385A', 'MEDAG', 'TRPM4'] |
| cytokine-mediated signaling pathway | 8,16E-04 | ['CCL13', 'CD40', 'CEBPD', 'LRRC3', 'PTGS2', 'IFIT1', 'SAMHD1', 'TNFSF13B', 'IFIT2', 'ICAM1', 'OASL', 'CCL8', 'CCL7', 'FLRT3', 'IL12A', 'JUNB', 'TRIM21', 'IL6R', 'RSAD2', 'TNFRSF12A', 'TNFSF15', 'MX1', 'IFNLR1', 'IRAK3', 'HLA-F', 'BATF', 'IL6', 'ZEB1', 'OAS1', 'RTN4RL1', 'IL7', 'IRF4', 'LEP', 'BCL2', 'IRF7', 'RHOU', 'IRF6'] |
| positive regulation of cell differentiation | 8,16E-04 | ['MEF2A', 'LRP5', 'AGT', 'MEDAG', 'TGFBR2', 'BMP4', 'TMEM100', 'MAPK11', 'BMP2', 'IL6', 'SFRP2', 'FRZB', 'ZC3H12A', 'SNAI1', 'JUNB', 'IL6R', 'ZNF385A', 'TRPM4'] |
| type I interferon signaling pathway | 4,73E-04 | ['RSAD2', 'OAS1', 'IRF4', 'MX1', 'IRF7', 'IRF6', 'IFIT1', 'HLA-F', 'SAMHD1', 'IFIT2', 'OASL'] |
| cellular response to type I interferon | 4,73E-04 | ['RSAD2', 'OAS1', 'IRF4', 'MX1', 'IRF7', 'IRF6', 'IFIT1', 'HLA-F', 'SAMHD1', 'IFIT2', 'OASL'] |

**Table S2.** Most enriched term analysis of differentially expressed gene in HEMn-LP after PIR silencing, which are potentially targeted by *JARID1B* transcriptional regulator.

| **Enriched term** | **FDR** | **Overlapping genes** |
| --- | --- | --- |
| transcription, DNA-templated | 2.94e-02 | AR,ATOH8,BCOR,BHLHE22,BNC1,COL4A2,EGR3,EYA4,FLI1,FOXD3,GLIS1,HEYL,HLX,JUN,MAF,MAML3,NFIX,NR4A2,PAX9,RARA,SIX1,SIX2,SOX10,SOX11,TBX18,TCF21 |
| regulation of cell population proliferation | 9.96e-05 | ADRA2A,AR,ATOH8,AVPR1A,BCL2,BMP2,BNC1,CDH13,EGR3,E2F1,FRZB,HLX,JUN,KCNK2,MYC,NGF,NRP2,NTF3,NTN1,PODN,PTPRU,RARA,SIX1,SIX2,SOX10,SOX11,SPEG,TGFBR3 |
| regulation of biosynthetic process | 3.32e-02 | AGAP2,AR,ATOH8,AVPR1A,BCL2,BCOR,BHLHE22,BMP2,BNC1,CDH13,EGR3,EYA4,FLI1,FOXD3,FST,GLIS1,HEYL,HLX,INHBB,JUN,KCNK2,MAF,MAML3,NFIX,NR4A2,PAX9,PTX3,RARA,RGMB,RIPK4,SIX1,SIX2,SOX10,SOX11,TBX18,TCF21,TGFBR3,TMEFF2 |
| anatomical structure morphogenesis | 1.00e-08 | AR,ATOH8,BCL2,BCOR,BMP2,CDH13,COL4A1,COL4A2,DSP,EGR3,EYA4,FJX1,FLI1,FOXD3,FRZB,GFRA1,GREB1L,HEYL,HLX,JUN,MMP15,NGF,NR4A2,NRP2,NTF3,NTN1,PAX9,RARA,RIPK4,SEMA7A,SIX1,SIX2,SLITRK5,SOX10,SOX11,TCF21,TGFBR3,TMEFF2,UNC5B |
| regulation of cell communication | 1.18e-02 | ADRA2A,AR,AVPR1A,BCL2,BICC1,BMP2,CDH13,CXXC4,EYA4,FRZB,FST,HEYL,INHBB,JUN,LUM,LYPD6B,MAML3,NGF,NR4A2,NSG1,NTF3,OXTR,PLK2,PODN,PTPRE,RARA,RTN4RL1,SEMA7A,SOX11,TBX18,TCF21,TGFBR3,THY1,UNC5B,WNT5B |
| cell differentiation | 8.54e-07 | AR,ATOH8,AVPR1A,BCL2,BHLHE22,BMP2,BNC1,COL4A1,COL4A2,DSP,EYA4,FLI1,FLNC,FOXD3,FRZB,FST,GFRA1,HEYL,HLX,INHBB,JUN,LUM,MAF,MFSD2A,MMP15,NEXN,NGF,NR4A2,NRP2,NTF3,NTN1,PLK2,PLXNC1,PTPRD,PTPRU,RARA,RTN4RL1,SEMA7A,SIX1,SIX2,SLITRK5,SOX10,SOX11,SPEG,TCF21,TGFBR3,THY1,UNC5B,WNT5B |

**CONSENT FOR THE CONDUCT OF THE INVESTIGATION PROJECT**

**Researcher / Clinical manager**:

**PROJECT TITLE:**

I …………………………………………………………………………………………………………………… with NID… .. ………………… I declare under my responsibility that I have read the Patient Information Sheet, of which a copy has been delivered to me. The characteristics and objective of the study have been explained to me, as well as the possible benefits and risks that I can expect, the rights that I can exercise, and the provisions on the treatment of data and samples. I have been given time and opportunity to ask questions, which have been answered to my satisfaction.

I know that my identity will be kept secret and that my samples will be identified with a coding system. I am free to revoke my consent at any time and for any reason, without having to give an explanation and without having a negative impact on any present or future medical treatment.

I consent to the use of my samples and associated data as part of this research project. I agree to participate voluntarily and I decline to claim any financial benefit for my participation in the study.

I hereby affirm that I have been warned about the possibility of receiving information regarding my health derived from the genetic analyzes carried out on my biological sample.

|  |
| --- |

I request information

|  |
| --- |

I do not want to receive information

once the research on the study results was completed.

If there was a surplus of the sample, I affirm that I was warned about the destination options at the end of the research project.

In this sense:

|  |
| --- |

1. I request the destruction of the excess sample

|  |
| --- |

1. I request that the excess sample be deposited at the Basque Biobank for Research-o + ehun

Date …………………… Signature of the patient ……………………………….

Date: ……………………. Signature of legal representative (if applicable) ………………..

Legal representative name:

Relationship with the patient:

I confirm that I have explained the characteristics of the research project and the conservation and security conditions that will be applied to the sample and the conserved data.

Name of the Investigator or the person designated to provide the information:

Signature …………………………… date………………………………
